# Development of Transgenic Minipigs with Expression of Antimorphic Human Cryptochrome 1

**DOI:** 10.1371/journal.pone.0076098

**Published:** 2013-10-16

**Authors:** Huan Liu, Yong Li, Qiang Wei, Chunxin Liu, Lars Bolund, Gábor Vajta, Hongwei Dou, Wenxian Yang, Ying Xu, Jing Luan, Jun Wang, Huanming Yang, Nicklas Heine Staunstrup, Yutao Du

**Affiliations:** 1 BGI-Shenzhen, Shenzhen, Guangdong, China; 2 BGI Ark Biotechnology, BGI-Shenzhen, Shenzhen, Guangdong, China; 3 ShenZhen Engineering Laboratory for Genomics-Assisted Animal Breeding, BGI-Shenzhen, Shenzhen, Guangdong, China; 4 Department of Biomedicine, University of Aarhus, Aarhus C, Denmark; 5 Central Queensland University, Rockhampton, Queensland, Australia; 6 Department of Biology, University of Copenhagen, Copenhagen, Denmark; 7 King Abdulaziz University, Jeddah, Saudi Arabia; Karlsruhe Institute of Technology, Germany

## Abstract

Minipigs have become important biomedical models for human ailments due to similarities in organ anatomy, physiology, and circadian rhythms relative to humans. The homeostasis of circadian rhythms in both central and peripheral tissues is pivotal for numerous biological processes. Hence, biological rhythm disorders may contribute to the onset of cancers and metabolic disorders including obesity and type II diabetes, amongst others. A tight regulation of circadian clock effectors ensures a rhythmic expression profile of output genes which, depending on cell type, constitute about 3–20% of the transcribed mammalian genome. Central to this system is the negative regulator protein Cryptochrome 1 (CRY1) of which the dysfunction or absence has been linked to the pathogenesis of rhythm disorders. In this study, we generated transgenic Bama-minipigs featuring expression of the Cys414-Ala antimorphic human Cryptochrome 1 mutant (hCRY1^AP^). Using transgenic donor fibroblasts as nuclear donors, the method of handmade cloning (HMC) was used to produce reconstructed embryos, subsequently transferred to surrogate sows. A total of 23 viable piglets were delivered. All were transgenic and seemingly healthy. However, two pigs with high transgene expression succumbed during the first two months. Molecular analyzes in epidermal fibroblasts demonstrated disturbances to the expression profile of core circadian clock genes and elevated expression of the proinflammatory cytokines IL-6 and TNF-α, known to be risk factors in cancer and metabolic disorders.

## Introduction

Upholding an entrained biorhythm is pivotal for the timing of metabolic processes (reviewed in [1], [2]). Hence, a highly developed hierarchical system of circadian clocks beginning with a master clock residing in the suprachiasmatic nucleus (SCN) of the anterior hypothalamus coordinates rhythmic cell behavior in all peripheral metabolic tissues [3], [4]. Cryptochrome (CRYs) proteins belong to a class of light-sensitive flavoproteins that play a core role in the molecular pathways underlying circadian rhythms in mammalian cells (reviewed in [1]). The heterodimeric transcription factor composed of the circadian locomotor output cycles kaput (CLOCK) and the brain and muscle aryl hydrocarbon receptor nuclear translator (ARNT)-like protein 1 (BMAL1) controls the expression of E-box containing genes including those coding for CRY and periodic circadian (PER) proteins. In turn, CRY/PER heterodimers inhibit CLOCK/BMAL1 transactivation activity through a negative transcriptional/translational feed-back loop (TTFL). Timely post-translational modifications of CRY and PER, such as phosphorylation by casein kinase I, target them for proteosomal degradation thereby releasing inhibition of CLOCK/BMAL1 in the auto-regulatory cycle [5], [6].

Regulation of several physiological systems including the endocrine, rest-activity cycle and metabolic system, is heavily intertwined with the circadian clock. In fact, 3–20% of all peripherally active genes are expressed with a periodicity of 24-hours and many of these are involved in metabolic processes [1], [7], [8], [9]. Naturally, recent evidence suggests that disturbance of circadian rhythms increases the risk of metabolic disorders such as obesity and type II diabetes [10], [11]. Moreover, mice with the sole modification of overexpressing an autologous but mutated CRY1 share key characteristics with type II diabetes mellitus patients [12].

Minipigs have become important biomedical models for human ailments due to the well described anatomical and physiological resemblance to humans. Hence, minipigs as an animal model for at least forty human ailments including cardiovascular and metabolic disorders have been described [13], [14], [15], [16].

Creation of transgenic animals by somatic cell nuclear transfer (SCNT) has been described for a variety of animals including sheep [17], mouse [18], [19], cow [20], [21] and pig [22], [23], [24]. In brief, the nuclei of genetically manipulated somatic donor cells are introduced into enucleated oocytes. Subsequently, the reconstructed embryos are implanted in the uterus of recipient sows. Recently a simplified procedure has been devised, termed handmade cloning (HMC) [25], [26], which has been applied in a number of cases including the generation of genetically engineered pigs [27]. Hence, minipigs expressing a mutated APP gene associated with early onset of Alzheimer's disease [28] have been produced by this method. More recently minipigs ectopically expressing human integrins as a model for skin inflammation [29], minipigs expressing a human PCSK9 gain-of-function mutant, as a model for early onset atherosclerosis [30], or minipigs expressing the nematode fat-1 gene for improved nutritional value of pigs [31] have also been produced.

Here we describe HMC-based generation of transgenic Bama-minipigs harboring an antimorphic hCRY1 mutation (Cys414-Ala), which previously has been shown to entail disturbance of the circadian rhythm but also phenotypes reminiscent of type II diabetes, such as hyperglycemia and polydipsia [32]. Twenty-three alive and healthy piglets were delivered and all animals presented peripheral exogenous Cry1 expression. Moreover, altered expression patterns of circadian clock and proinflammatory genes were evident in cultured fibroblasts, suggesting a disturbance of peripheral oscillators.

## Materials and Methods

### Ethics statement

Animal experiment procedures were approved by the life ethics and biological safety review committee of BGI-Research.

### Plasmids and hCry1^AP^ vector construction

The CMV promoter in pcDNA3.1 (Invitrogen, Paisley, UK) was released by BglII/NheI and exchanged with the CMV early enhancer and chicken beta-actin (CAG) promoter PCR amplified from pEGFP-N1 (Clontech, CA, USA). The new construct was denoted pCAG-Neo. A multiple cloning site was, subsequently, inserted in EcoRI and NotI relaxed pCAG-Neo. The first intron of the chicken beta-actin gene was inserted into NheI/EcoRI. Human Cry1 (hCry1) cDNA was purchased from FulenGen (ID:M0114, FulenGen, Guangzhou, GD, China). The Cys414-Ala mutation was introduced by site-directed mutagenesis (QuikChange, Stratagene, Santa Clara, CA, USA) employing 5′-CAGTTTTTTCACTGCTATGCCCCTGTTGGTTTTGGTAGG-3′ and 5′-CCTACCAAAACCAACAGGGGCATAGCAGTGAAAAAACTG-3′ as forward and reverse PCR primers, respectively. The PCR product was digested with DpnI and inserted into the pMD18-T plasmid (TaKaRa, Shiga, Japan) and subsequently transformed into competent DH5α cells. The purified plasmid was digested with AgeI/NotI to release the hCry1^AP^ CDS fragment, which was then inserted into EcoRI/NotI digested pCAG-intron-Neo producing the final construct designated pCAG-intron-hCry1^AP^.SV40-Neo.

### Generation of transgenic donor cells for HMC

Porcine fetal fibroblasts (PFF) derived from a 32 d old Bama (BM) pig fetus were cultured in DMEM (Gibco, Invitrogen, Paisley, UK) supplemented with 15% FBS (HyClone, Logan, UT, USA), 1% L-glutamine, and 1% NEAA and maintained in 5% CO_2_ atmosphere at 37°C. Approximately 1.5×10^6^ BM-PFF cells were transfected, using an Amaxa Nucleofector kit (Lonza, Verviers, Belgium) according to manufacturer's directions and seeded into 6-well cell plates (JET Biofil, Guangzhou, China). One day after transfection, cells were trypsinized and reseeded into six 10 cm culture dishes (Becton Dickinson, Lincoln Park, NJ, USA) in complete DMEM medium containing 500 µg/mL G418 (Invitrogen). After 10 days of drug selection, G418-resistant colonies were picked and expanded.

### Handmade cloning and embryo transfer

The specific HMC procedure used for pig cloning has previously been described [25], [31]. Briefly, cumulus–oocyte complexes (COCs) collected from porcine ovaries were washed and incubated in 4-well plates for *in vitro* maturation (IVM) at 38.5°C in 5% CO_2_ humidified atmosphere. After 41–42 h cumulus cells were separated from the matured oocytes by hyaluronidase treatment. Following partial digestion of the zonae pellucidae, nuclei were removed by bisection. Each cytoplast was transferred into a fusion chamber containing fusion medium and fused to a single transgenic fibroblast with a single 100 V direct current (DC) impulse of 2.0 kV/cm for 9 µs. After one hour, each cytoplast-fibroblast pair was fused with another cytoplast in activation medium using a DC impulse of 0.8 kV/cm for 80 µs. Hereafter, the fused cells were incubated for 4–6 h in porcine zygote medium 3 (PZM-3) supplemented with 5 µg/ml cytochalasin B and 10 µg/ml cyclohexinmide at 38.5°C, 5%CO_2_, 5% O_2_, and 90% N_2_ at maximum humidity. Reconstructed embryos developed into transgenic blastocysts during an additional 6 days of culture in PZM-3 medium and were eventually transferred surgically into the uterine horns of recipient sows [33]. Pregnancies were diagnosed by ultrasonography on day 28 post-transfer and monitored every 2 weeks afterwards [25]. Twenty-three live, transgenic minipigs were eventually obtained.

### Identification of hCry1 in the transgenic animals

Genomic DNA was phenol-chloroform extracted from transfected primary fetal fibroblasts or tail clips from the 23 transgenic minipigs and a non-transgenic control (#321-1^wt^) at seven days of age. Transgene identification was carried out in PCR reactions of 20 ng genomic DNA, 20pmol of each primer (cACTB intron forward 5′-TTCATACCTCTTATCTTCCTCCCA-3′ and CRY1 reverse 5′-CTTCCACTGCTGCTACAACCTG-3′) and 0.5 unit of rTaq polymerase (Takara). PCR conditions were as follows; 5 min at 95°C; 35 cycles of 30 sec. at 95°C, 30 sec. at 60°C, 40 sec. at 72°C; and final extension at 72°C for 5 min. Finally, 4 µL PCR product was electrophoresed on a 1.0% agarose gel.

For relative measurements of hCry1^AP^ mRNA levels total RNA was Trizol extracted from transfected primary fetal fibroblasts as well as from tail clips of the 23 transgenic minipigs and the non- transgenic control (#321-1^wt^). Subsequently, 1 µg RNA was in one step DNase treated and reverse transcribed using the RevertAid First Strand cDNA Synthesis Kit (Takara) according to manufacturer's instructions. From the total cDNA, 1 µL was used for quantification using an ABI 7500 Real-Time PCR machine (ABI, CA, USA), Power SYBR Green (Takara) and hCry1 specific primers (**Table 1**). Conditions were as follows: 30 sec. at 95°C; 35 cycles of 5 sec. at 95°C, 34 sec at 60°C, and 40 sec. at 72°C. GAPDH was used as internal control (**Table 1**).

**Table 1:** Quantitative RT-PCR primer-sequences.

Gene name	Forward primer	Reverse primer
**pPer2**	ACACCCAGAAGGAGGAGCAGAGC	CGAGGCTTGACCCGTTTGGACTT
**pBaml1**	TTTGTCGTAGGATGTGACCGAGGGA	CGCCGTGCTCCAGAACATAATCG
**pCry1**	CTTCTTGCGTCAGTGCCATCTAA	ATGATGCTCTGCGTGTCCTCTTC
**hCry1**	TTCATACCTCTTATCTTCCTCCCA	CTTCCACTGCTGCTACAACCTG
**pTNF-α**	CCACGCTCTTCTGCCTACTG	GAGGTACAGCCCATCTGTCG
**pIL-6**	AGGGAAATGTCGAGGCTGTG	CTCAGGCTGAACTGCAGGAA
**pClock**	GAACAATAGACCCAAAGGAACCA	CCCAGAACTTCAAATGGCAAATA

### Transcription analysis of cytokine and circadian clock genes

Epidermal fibroblasts from three transgenic pigs (#175-3^AP^, #175-5^AP^, and #377-3^AP^) and a non-transgenic pig (#321-1^wt^) were expanded from ear-derived skin biopsies. The established cell cultures were seeded in 12-well plates and maintained until 100% confluency at which the cells were synchronized with 50% FBS in DMEM for 2 hours and successively recovered in serum-free medium for 6 hours. At the given time points the cells were harvested and total RNA Trizol extracted. Quantitative RT-PCR for the gene-transcripts of porcine Per2, Cry1, Clock, Bmal1, Interleukin-6 (IL-6), Tumor-necrosis-factor alpha (TNF-α), and the transgenic human Cry1 (**Table 1**) was performed as described above.

### Body temperature measurements

Three transgenic (#208-2^AP^, #377-1^AP^, #377-6^AP^) and three non-transgenic (#321-1^wt^, #321-2^wt^, #321-3^wt^) minipigs were housed individually with 11:13 h light-dark conditions. They were fed three times a day (8:00 am, 2:00 pm, and 6:00pm) with a standard swine feed and had unlimited access to water. Body temperature was measured using an infrared thermometer (DT-8806H, CEM, Hong Kong) every second hour through 98 hours.

### Statistical analysis

P-values were calculated by a two-tailed Student's t-test or a one-sided ANOVA where appropriate to test the null hypothesis of no difference between the compared groups. The assumption of equal variances was tested by F-test; if significantly different Welch's correction was applied. The assumption of equal SD was tested by Bartlett-test; if significantly different the non-parametric Dunn's Multiple Comparisons Test was used. Assumption of normality was tested using the Kolmogorov and Smirnov method. In all statistical analyses, p-values <0.05 were considered significant.

## Results

### Establishment of transgenic donor cells for HMC

**Figure 1:**
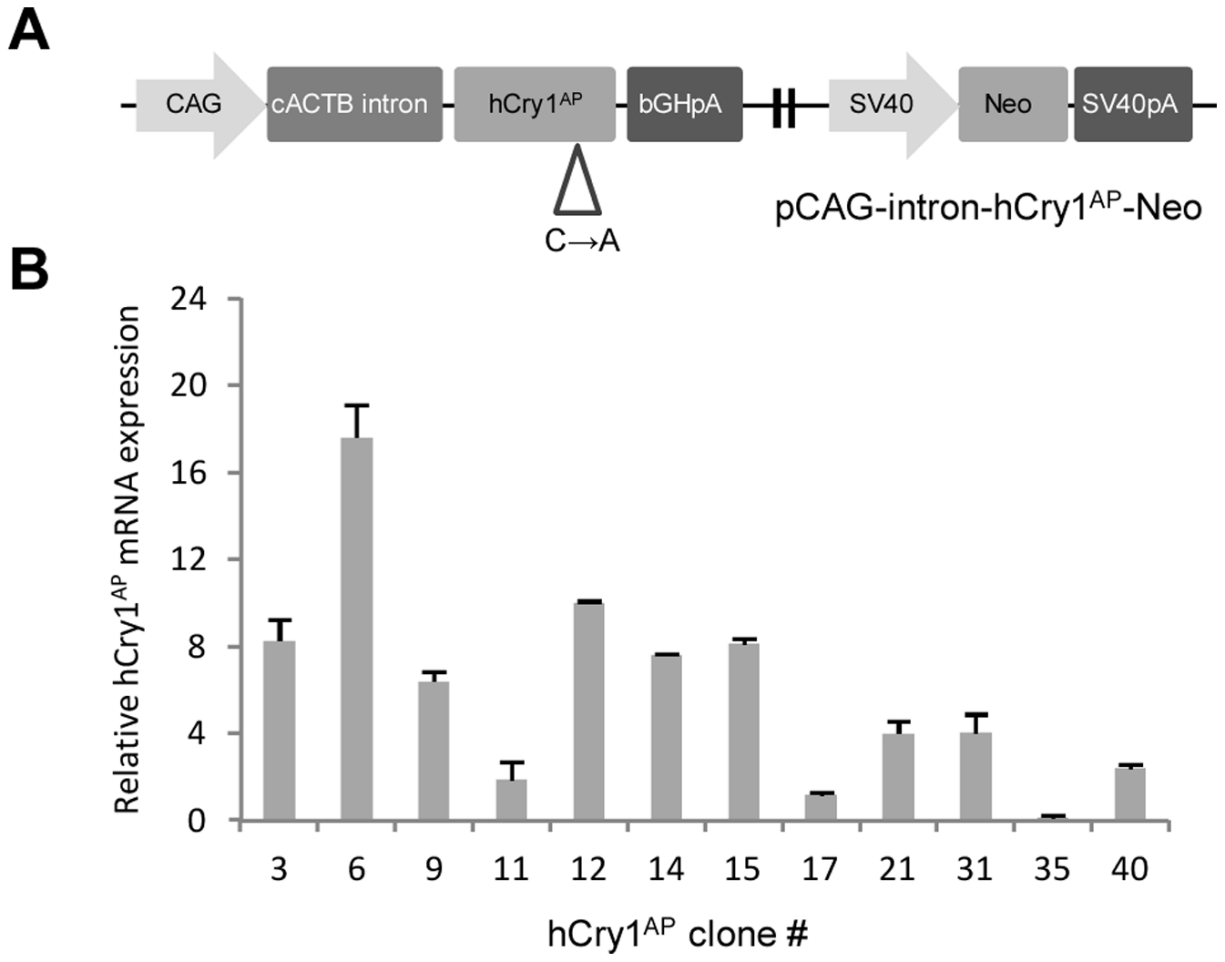
Functional analysis of the antimorphic human Cry1 expression vector. (**A**) Graphic illustration of the pCAG-intron-hCry1^AP^.SV40-Neo expression vector. Expression of the antimorphic human Cry1 (hCry1^AP^) is driven by the CMV enhancer chicken beta-actin (CAG) chimeric promoter and terminated with the bovine growth hormone polyadenylation site (bGHpA). A selection cassette consisting of a Simian virus 40 (SV40) promoter, a Neomycin (Neo) gene and SV40 polyadenylation signal is placed downstream. (**B**) Quantitative RT-PCR analysis of hCry1^AP^ expression in transgenic Bama-minipigs fetal fibroblasts (BM-PFFs). Reverse transcribed total RNA was used for amplification with hCry1-specific exon-exon primers and normalized to endogenous GAPDH. The depicted expression levels are relative to cell clone # 17. The experiment is performed in triplicate and data are presented as mean values ± standard deviation.

With the objective to selectively express an antimorphic human CRY1 (hCRY1) mutant in cultured cells, we firstly constructed a vector comprising two modules; (i) a CAG-intron-hCry1^wt^ expression cassette composed of the CAG promoter, the first intron of chicken beta-actin (cACTB) gene and the wt hCRY1 gene, and (ii) a downstream SV40-Neo selection cassette composed of the SV40 promoter and the neomycin (Neo) resistance gene. Hereafter, the hCry1 Cys414-Ala antimorphic mutation (hCry1^AP^) was introduced by site-directed mutagenesis generating the final vector pCAG-intron-hCry1^AP^.SV40-Neo (**Figure 1A**). Cys414 is part of a well conserved CP dipeptide motif across CRY-proteins and phyla, indicating its importance for CRY1 protein functionality. Based on the solved crystal-structure of murine CRY2, the CP motif appears situated within a co-factor binding pocket regulating CRY1 stability through the interaction with the ubiquitin ligase complex SCF^FBXL3^, flavin adenine dinucleotide (FAD) and PERs [34]. Although both the catalytic function with PER proteins and repression of the CLOCK/BMAL1 complex appears unaffected by the Cys414-Ala point mutation [12], [32], circadian rhythms and cellular function (in e.g. pancreatic beta-cells) are disturbed. Thus, suggesting that Cry1^AP^ affects kinetic and dynamic properties or influences non-canonical circadian pathways.

In order to generate hCRY1^AP^ expressing donor cells, we stably transfected Bama porcine fetal fibroblasts (BM-PFFs) with pCAG-intron-hCry1^AP^.SV40-Neo and applied a selection pressure with G-418 for 10 days. Subsequently, 25 individual G-418 resistant colonies were isolated and expanded. Genomic DNA extracted from the 25 clones was subjected to PCR analysis with Cry1-Neo specific primers revealing 12 positive clones (data not shown). Subsequently, RT-qPCR on total mRNA extracted from the 12 positive clones specific for hCry1 transcripts demonstrated that hCry1 was expressed in all clones at varying intensities (**Figure 1B**). Thus, relative to hCry1^AP^ clone # 35, the three clones designated # 6, # 12 and # 15 exhibited the highest expression levels, with hCry1 mRNA levels 17, 10 and 8 fold above that of clone # 35, respectively. These high-expressing clones were chosen for HMC. Importantly, the hCry1 transcript was undetectable in un-transfected BM-PFFs.

### Generation of transgenic cloned pigs by HMC

**Figure 2:**
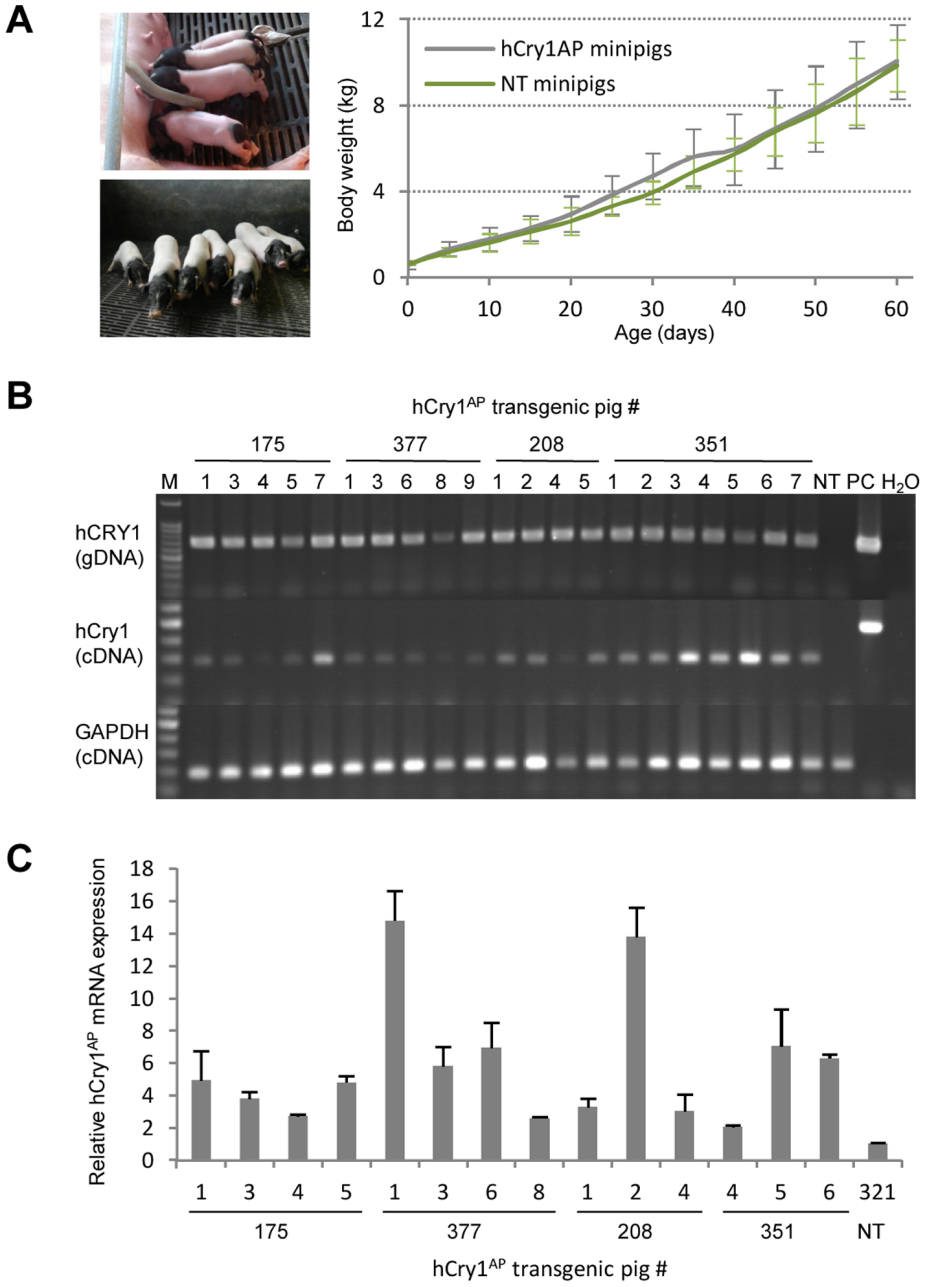
Demonstration of transgenesis in cloned minipigs produced by HMC. (**A**) Pictures of cloned Bama-minipigs at the age of five and 60 days. Curve indicates mean weight increase over the first 60 days of the 21 transgenic (hCry1^AP^) and five non-transgenic (NT) animals (#321-1^wt^, #321-2^wt^, #321-3^wt^, #321-5^wt^, and #321-6^wt^). (**B**) PCR and RT-PCR analysis on gDNA and total RNA, respectively, isolated from tail clips of the 23 cloned minipigs born from three recipient sows (#175, #377 and #208) as well as from one non-transgenic (NT) control. The PCR analysis employing hCry1 specific primers revealed genomic integration of the transgenic cassette in all the cloned animals (upper panel). RT-PCR analysis using exon-exon primers for hCry1 and porcine GAPDH showed robust expression of hCry1^AP^ in the transgenic animals with no detectable band in the lane corresponding to the NT control (lower two panels); PC, plasmid control; M, 100 bp marker. (**C**) Quantitative RT-PCR performed on cDNA from 14 of the 23 transgenic animals and a NT control (#321-1^wt^). Total mRNA extracted from tail clips was reverse transcribed and used for quantification of hCry1 normalized to endogenous ACTB. The expression values are relative to the NT control. The experiment is performed in triplicate and data are presented as mean values ± standard deviation.

The selected hCry1^AP^ clones (# 6, # 12 and # 15) were individually applied as donor cells for HMC. The blastocyst rate was 45.5%, and a total of 691 reconstituted embryos were surgically transferred to the uteri of six naturally cycling recipient sows, with 90–135 reconstituted embryos transferred per recipient (**Table 2**). Of the six recipient sows, five became pregnant from which four went to term delivering a total of 32 piglets of which six were stillborn and two were mummified. Of the living 24 piglets one presented with malformations and was euthanized. Further two succumbed within the first months. The surviving 21 piglets remain healthy and gain weight according to the growth curve of the herd (**Figure 2A**).

**Table 2:** Summary of cloning efficiencies obtained with hCRY1^AP^-transgenic fibroblasts.

Donor sow ID	Blastocysts transferred	Donor cell	Delivered piglets	Alive	Presenting malformations	Stillborn	Mummified
**S1-377**	135	hCRY1^dn^-6	9	5		2	2
**S2-351**	111	hCRY1^dn^-6	10	8		2	
**S3-175**	135	hCRY1^dn^-12	7	6		1	
**S4-208**	134	hCRY1^dn^-12	6	4	1	1	
**S5-584**	93	hCRY1^dn^-15	Not pregnant				
**S6-180**	83 (17PA)	hCRY1^dn^-15	Abortion				
**Total**	691		32	23	1	6	2

### Identification of hCry1^AP^ expression in the twenty-three transgenic minipigs

All cloned minipigs were evaluated for transgene integration and expression. Hence, transgenic status was demonstrated in all 23 cloned animals by PCR amplification of hCry1 on tail clip extracted genomic DNA obtained from the cloned minipigs at the age of one week. A PCR fragment with the expected size of 746 bp appeared in the lanes for each of the cloned minipigs but not for an age-matched non-transgenic (NT) control (**Figure 2B** **, upper**). Assessment of transgene expression was achieved by conducting RT-PCR on total RNA extracted from the tail clips. Again an easily detectable band corresponding to hCry1 cDNA was observed for all transgenic minipigs but not for the NT control (**Figure 2B** **, lower**).

Analysis of the expression level of Cry1 showed that the relative expression level in the transgenic minipigs varied up to 7-fold compared to that in the lowest expressing transgenic animal (#351-4^AP^) (**Figure 2C**). A general higher expression level was detected in animals originating from donor cell # 6 (sow #377 and #351) suggesting a correlation between the expressions observed in the donor cells and in the transgenic minipigs. Interestingly, the expression appears to be more heterogeneous in piglets arising from donor cell # 6 compared to the expression pattern among piglets originating from donor cell # 12 (sow #175 and #208). This suggests that non-inherited positional effects work on the transgene - especially in animals originating from donor cell # 6. Pig #377-1^AP^ and pig #208-2^AP^ were the animals displaying the highest relative hCry1^AP^ expression. Notably, they perished at the age of about 35 days, hinting a possible concentration dependent toxicity.

### Altered oscillation of circadian gene expression in the transgenic minipigs

**Figure 3:**
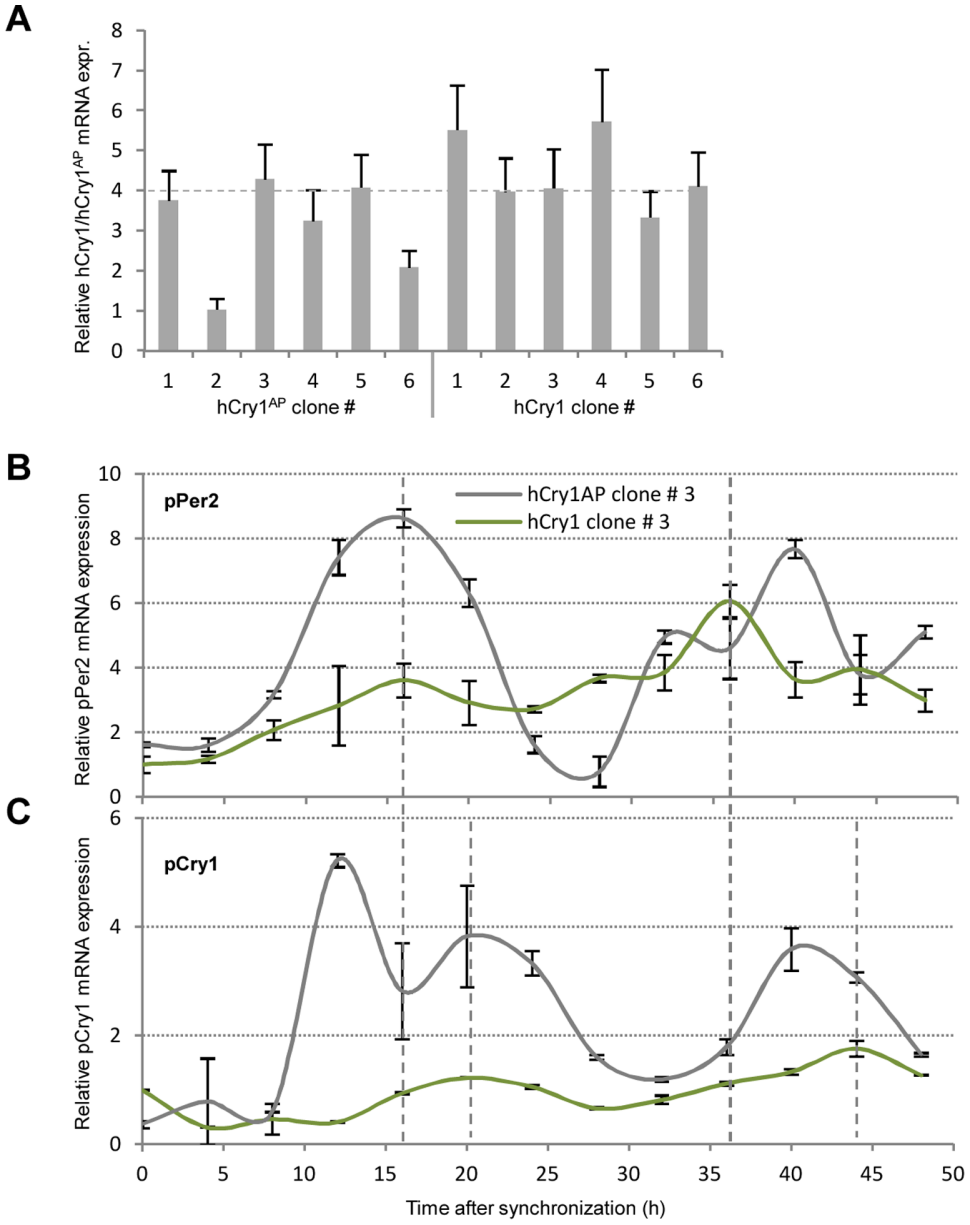
Expression of the antimorphic but not the wt hCry1 instigate altered expression profiles of pPer2 and pCry1. Primary porcine fibroblasts were stably transfected with pCAG-intron-hCry1.SV40-Neo or pCAG-intron-hCry1^AP^.SV40-Neo and relative expression were assessed by RT-qPCR with exon-exon spanning hCry1 primers. (**B–C**) Serum-shocked cells were harvested every fourth hour through 48 hours. Total RNA was extracted and used for RT-qPCR with exon-exon spanning primers targeting pPer2 or pCry1. GAPDH normalized data relative to hCry1 clone # 3 is depicted as a function of time. The experiment is performed in triplicate and data are presented as mean values with smoothened curves. Grey dashed lines indicate the first and second zenith of pPer2 and pCry1 mRNA expression in hCry1 containing cells.

Clock-gene expression levels inherently oscillate in well defined phases with time in intact peripheral tissue. In cell culture this feature is lost but can, however, be reinitiated and synchronized through serum-shock. Hence, in *ex vivo* fibroblasts this leads to a cell-autonomous oscillation in clock gene expression for at least 48 hours [35], [36]. Furthermore, functional CRY1 is required for a sustained circadian rhythm in dissociated murine fibroblasts [37]. In order to evaluate if the introduced point mutation in hCry1 would exert disruption to the cell-autonomous oscillation, we transfected primary porcine fibroblasts with a construct expressing the functional hCRY1 protein (pCAG-intron-hCry1.SV40-Neo) or the antimorphic variant (pCAG-intron-hCry1^AP^.SV40-Neo). Following antibiotic selection and RT-qPCR analysis, clones expressing the functional (clone # 3) or antimorphic hCry1 (clone # 3) at comparable intensity and displaying similar growth rates were selected and further expanded (**Figure 3A**). At confluency the cells were serum shocked for 2 h after which they were returned to serum-free medium for an additional 6 h. Subsequently, cells were harvested for RT-qPCR analysis every 4 hours through 48 hours.

The mRNA level of pPer2 in the hCry1 transfected cells appears to have a first zenith at Zeitgeber time (ZT) 16 h and a second at ZT 36 h (**Figure 3B**). On the other hand, the zeniths of pCry1 mRNA expression are slightly offset occurring at ZT 20 h and 44 h (**Figure 3C**). This slight offset but synchronous oscillation of Per2 and Cry1 has previously been documented in SCN as well as peripheral tissue [38]. Interestingly, the rhythmic pattern is only partially maintained in the hCry1^AP^ transfected cells and, moreover, displays oscillations of greater magnitude, suggesting that the regulation of pPer2 and pCry1 is unbalanced in the presence of the antimorphic hCRY1^AP^.

**Figure 4:**
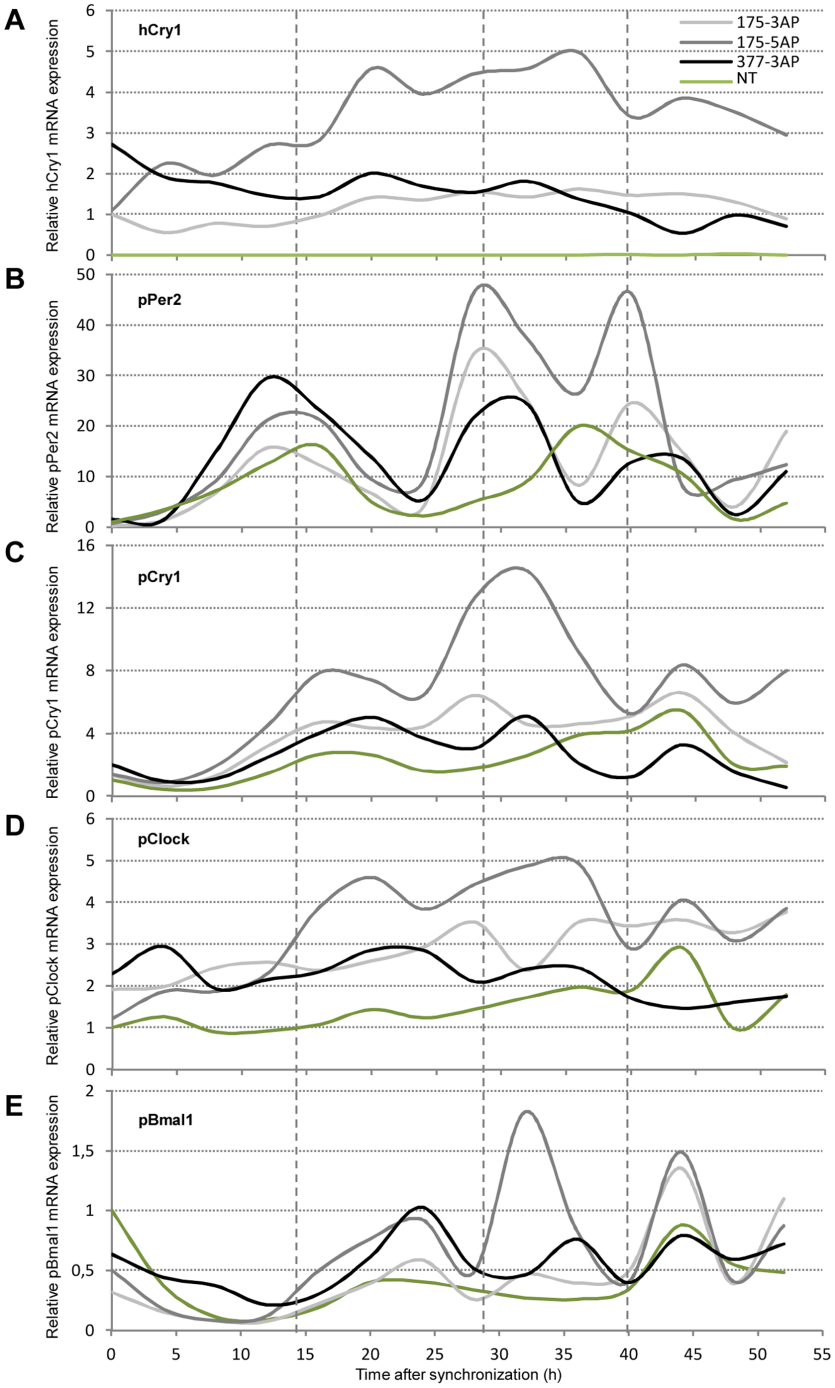
Altered oscillation patterns of key clock transcripts in fibroblasts from transgenic minipigs. Fibroblasts were expanded from skin-biopsies obtained from three transgenic minipigs (#175-3^AP^, #175-5^AP^, and #377-3^AP^) and one NT control (#321-1^wt^). The cells were serum-shocked after which total RNA was extracted every fourth hour through 52 hours. Quantitative RT-PCR was performed with exon-exon spanning primers targeting (**A**) hCry1 (**B**) pPer2 (**C**) pCry1 (**D**) pClock and (**E**) pBmal1 normalized to endogenous ACTB. Expression is relative to minipig #175-3^AP^ in (**A**) and to the NT control in (**B–E**) and is depicted as a function of time. The experiment is performed in triplicate and data are presented as mean values with smoothened curves. Grey dashed lines indicate the first, second and third zenith of pPer2 mRNA expression in cells from minipig #175-5^AP^.

With the intent to examine circadian gene expression patterns in NT as compared to transgenic minipigs we cultured epidermal fibroblasts from transgenic minipigs (#175-3^AP^, #175-5^AP^ and #377-3^AP^) and from a NT control (#321-1^wt^). At confluency the cells were serum shocked for 2 h and then allowed to recover in serum-free medium for another 6 h. Afterwards the cells were harvested for RT-qPCR analysis every 4 hours through 52 hours. Firstly, we validated hCry1^AP^ mRNA expression in all the three cell populations. After an initial increase in expression level in pig #175-5^AP^, all mRNA levels (relative to pig #175-3^AP^) remained within a stable range with only minor yet rhythmic fluctuations. Importantly, no call above background was observed in the control (**Figure 4A**). Secondly, we examined porcine Cry1, Per2, Clock and Bmal1 mRNA levels in the cultured cells following synchronization. Given the nature of the feedback regulatory effect of CRY1/PER2 on CLOCK/BMAL1 the corresponding two sets of genes are expected to have peak expression levels occurring in antiphase to each other under normal entrainment. In fibroblasts from the NT control pig #321-1^wt^, pPer2 and pCry1 transcript levels displayed around 24 h oscillations with a first zenith around ZT 15 h for pPer2 and ZT 17 h for pCry1 (**Figure S1**). Notably, the oscillations of Per2 and Cry1 appeared slightly displaced and with a circadian rhythm similar to the one observed in the transfected porcine fibroblasts. Conversely pBaml1 transcript levels displayed a first nadir around ZT 12 and 36, thus, being inversely correlated to pPer2 and, to a lesser extent, pCry1 expression. On the other hand, pClock mRNA levels presented a much weaker circadian pattern although a weak zenith is detectable a ZT 20 and 44. However, this seems to be consistent with previous findings in murine peripheral tissues [39]. In the transgenic animals the expression curves of hCry1, pPer2, pCry1, pClock, and pBmal1 displayed key differences and similarities compared to the NT control (**Figure 4 A–E**). Oscillation in the mRNA levels of hCry1 follows that of pCry1, indicating that hCry1 is susceptible to the same post-transcriptional regulation otherwise governing the levels of pCry1. Supporting this notion, is the observation that the 3′ untranscribed region (UTR) of mouse Cry1 mRNA contains a destabilizing element to which heterogeneous nuclear ribonucleoprotein D (hnRNP D) binds facilitating mRNA degradation [40]. As in the NT control, expression levels of pPer2, pClock and pBaml1 in the transgenic animals follow a rhythmic pattern. However, in the latter the amplitude is increased and the oscillation period decreased. Hence, the ZT for the first zenith is comparable between the control and the transgenic animals. However, cells from the latter seem to complete two cycles within 24 h compared to a single cycle in the NT control.

Group means and analysis of variance between group means at each time point is summarized in **Table S1 and S2**.

Looking specifically at the acrophases of pCry1 and pPer2 within the first 24 hours, there is a high concordance between the cells derived from the transgenic and NT animals (**Figure S2**). Hence for pCry1 the acrophase is at 18.9 h (±0.6 h) in the transgenic cells and at 18.4 h in the NT control cells. Similar, for pPer2 the acrophase occurred at 14.1 h (±0.2 h) in the transgenic cells and at 13.6 h in the NT control cells. This rhythmicity is maintained in the NT cells producing pCry1 and pPer2 acrophases on day 2 at 16.4 h and 13.6 h, respectively. Interestingly, however, data from the transgenic cells only fitted the applied model if subdivided into 12 h time frames. Giving acrophases at 7 h (±0.2 h) and 19.7 h (±1.2 h) for pCry1 and 5.8 h (±0.2 h) and 16.4 h (±1.4 h) for pPer2. Taken together, the redundancy in the TTFLs seemingly ensures rhythmicity in the transgenic fibroblasts but with an altered circadian period (τ) and amplitude. A slight intra-individual variation in τ seems evident but falls in line with previous reports on circadian rhythms in human fibroblasts [41].

### Increased activation of proinflammatory markers in the hCry1^AP^ transgenic minipigs

**Figure 5:**
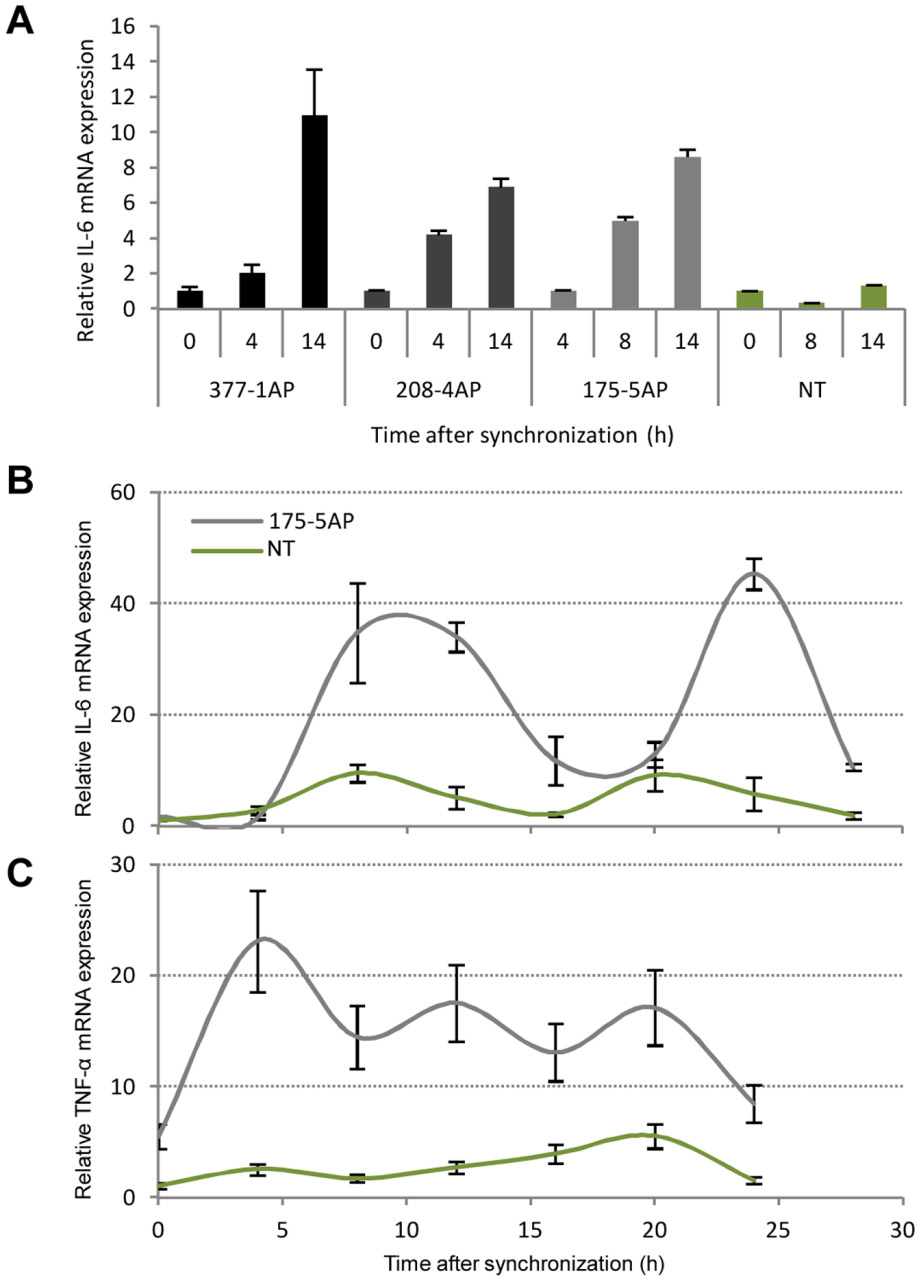
Induction of the proinflammatory cytokines IL-6 and TNF-α in fibroblast from the hCry1^AP^ transgenic animals. (**A**) Fibroblasts derived from skin-biopsies obtained from three transgenic minipigs (#377-1^AP^, #175-5^AP^, and #208-4^AP^) and one NT control (#321-1^wt^) were serum-shocked and allowed to recover. Subsequently, total RNA was extracted at three time points within 14 hours post-recovery. Quantitative RT-PCR was performed with exon-exon spanning primers targeting porcine IL-6 and normalized to endogenous ACTB. Values are relative to the first measurement time point. (**B**) Relative IL-6 RT-qPCR on total RNA extracted from serum-shocked fibroblasts originating from transgenic minipig #175-5^AP^ and NT control pig #321-1^wt^ as above. Cells were harvested every fourth hour through 24–28 hours. Values are relative to the first measurement time point (ZT 0). (**C**) Relative TNF-α mRNA expression in fibroblasts from transgenic minipig #175-5^AP^ and NT control pig #321-1^wt^ as above. All experiments are performed in triplicate and data are presented as mean values ± standard deviation.

Next we wanted to determine whether the altered circadian rhythms and/or the expression of the antimorphic hCry1 in the transgenic animals would lead to activation of proinflammatory cytokines. The level of IL-6 and TNF-α mRNA was quantified in cDNA originating from serum shocked hCry1^AP^ transgenic fibroblasts (derived from pig #377-1^AP^, #208-4^AP^, and #175-5^AP^) and NT fibroblasts (derived from pig #321-1^wt^). An initial analysis revealed a sharp and linear increase in the mRNA level of IL-6 in the transgenic animals reaching a 7–10 fold increment at ZT 14 compared to ZT 0. Notably, this observation was absent in the NT control (**Figure 5A**). To confirm these findings we repeated the experiment with epidermal fibroblasts derived from pig #175-5^AP^ and a NT control (#321-1^wt^) with measurements every fourth hour through 28 hours (**Figure 5B**). The more detailed time course confirmed the initial steep increase in IL-6 mRNA levels until ZT 12 after which the level dropped off reaching a nadir at ZT 20. Comparing the expression levels of IL-6 at nadir and zenith, minipig #175-5^AP^ presents a level about 3-fold higher relative to the NT control. Notably, however, the IL-6 mRNA level in both the transgenic and NT animal revolted with a τ of approximately 12 hours. In a parallel experiment assessing TNF-α mRNA levels in the same pool of cells derived from pig #175-5^AP^ and the NT control showed significant increase in TNF-α expression in the transgenic minipig, that is, reaching levels 3–9 fold higher relative to the levels observed in the control (**Figure 5C**). This lends further support to the notion of proinflammatory induction in the transgenic cells. Interestingly, a complete circadian cycle was completed in 16 hours in cells from the NT control animal whereas in only took 8 hours for a complete cycle in the cells derived from the transgenic animal.

### Circadian oscillation of body temperature

**Figure 6:**
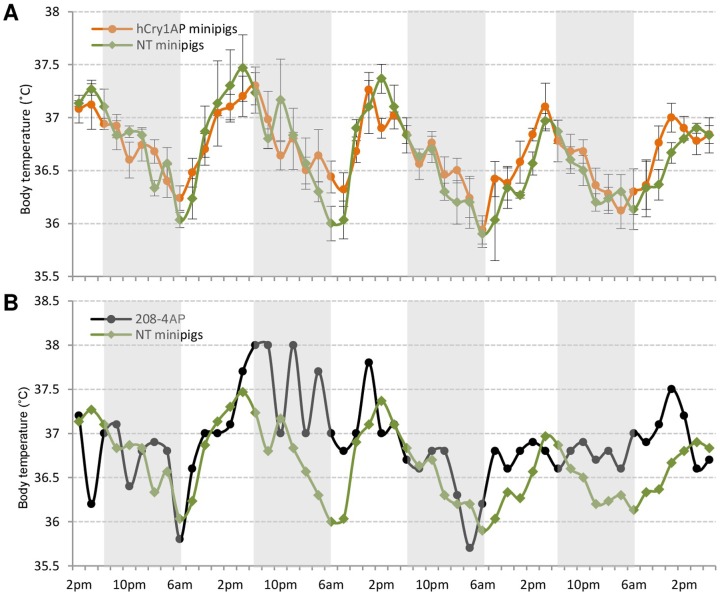
Discrete circadian oscillation of body temperature in hCry1^AP^ transgenic compared to NT control minipigs. (**A**) Body temperature was measured using an infrared thermometer in five transgenic minipigs (#175-5^AP^, #377-2^AP^, #351-3^AP^, #351-6^AP^, and #208-4^AP^) and three NT control animals (#321-1^wt^, #321-2^wt^, and #321-3^wt^) every second hour over a time course of 4 days. Mean body temperature is plotted as a function of time ± standard deviation. (**B**) Body temperature fluctuations of minipig #208-4^AP^ compared to the mean from the three NT control minipigs (#321-1^wt^, #321-2^wt^, and #321-3^wt^) obtained as described above.

In mammals, body temperature (T_b_) shows circadian oscillation. Therefore, recording T_b_ fluctuation is considered an effective way of determining circadian rhythm perpetuations [42]. Body temperature was recorded every second hour through 4 days in five transgenic minipigs (#175-5^AP^, #208-4^AP^, #351-3^AP^, #351-6^AP^, and #377-2^AP^) and three NT minipigs (#321-1^wt^, #321-3^wt^, and #C-28^wt^) (**Figure 6A**). Animals in both groups displayed normal T_b_ curves with a steady increase during daylight and a steady decrease during nighttime. Three of the five hCry1^AP^ and two of the three NT pigs showed rhythmic T_b_ oscillations with τ around 23 hours as calculated by the chi-square periodogram (**Table S3**). Among the remaining three animals, especially the transgenic minipig #208-4^AP^ exhibited an abnormal T_b_ curve (**Figure 6B**). In summary, these results may imply that expression of the antimorphic mutant hCry1^AP^ potentially leads to disturbance of the circadian rhythm of T_b_ in the transgenic minipigs.

## Discussion

Minipigs have become an attractive mammalian model for research in human ailments including metabolic, cardio-vascular and degenerative disorders. Small body sizes as well as physiological and anatomical resemblance to humans has raised the interest in minipigs and so basic as well as clinical studies have already been conducted. Furthermore, the complete sequencing of the pig genome has further spawned a general interest in genetically modified porcine disease-models [43]. In this report we describe the generation of Bama-minipigs holding a genomically integrated transcription cassette from which a heterologous and antimorphic CRY1 is expressed. The transgene is under the control of a CAG promoter which has shown almost ubiquitous activity in all tissues including the brain and skin [44], [45]. Expression of the transgene in the 23 live-born, seemingly healthy, cloned animals was verified by PCR and RT-qPCR on DNA and RNA, respectively, extracted from skin biopsies. Curiously, despite originating from only two different donor-cell populations the expression pattern in the transgenic minipigs appeared rather heterogeneous. As the cycle of the external cues (light and feed) are highly controlled it is unlikely that the circadian clocks among the pigs are out of synchronization. However, stochastic events, genomic rearrangements or epigenetic changes during embryogenesis could explain the variation in expression.

Next we wanted to assess to what extent the introduced antimorphic hCry1^AP^ would be expressed in cultured fibroblasts from the pigs and whether this would alter the autonomous peripheral oscillations of other key clock components. It is well known that several clock effectors including Per2 and Cry1 contain 3'UTRs prone for mRNA degradation [40], [46]. Interestingly, the mRNA level of hCry1^AP^ itself oscillated with a periodicity comparable to pCry1, suggesting that the transgenic Cry1 is subject to the same post-transcriptional regulation as the endogenous Cry1. Furthermore, there appears to be consistency between expression levels of hCry1^AP^ and the clock genes, hence, minipig #175-5^AP^ presented the highest relative expression in all assays.

Whereas peripheral oscillations of core clock gene transcripts demonstrated an approximately 24 hours periodicity in epidermal fibroblasts from the NT control pig, the same gene transcripts oscillated with a frequency of about 12 hours in cells from the three transgenic animals tested (#175-3^AP^, #175-5^AP^, and #377-3^AP^). Importantly, previous reports demonstrated coherence between peripheral oscillations *in vitro* and *in vivo*. Furthermore, deviations in the circadian rhythm of clock gene expression were found to be retained *in vitro* [47], [48]. These results, thus, appear to be in line with the abnormal circadian behavior observed in mouse Cry1^AP^ transgenic mice [32]. This indicates that the regulatory function of CRY1 cannot be rescued by CRY2 or a second regulatory loop, involving the orphan nuclear receptors Rev-Erbα and RORα.

Oscillations in the mRNA levels of the clock driving genes pClock and pBaml1 are quantitatively small compared to those of the regulatory arm, pCry1 and pPer2. Importantly, however, there is an anti-phase correlation between the former and pPer2, suggesting that regulation of the cell-autonomous circadian clock is maintained but with a τ of only 12 hours. Notably, oscillation of pPer2 is both very consistent and robust across all transgenic fibroblast cultures. This supports not only the notion of pPer2 being the primary intrinsic Zeitgeber of rhythmicity in peripheral tissue [49], [50] but also substantiating the τ alternating effect of hCRY1^AP^. Furthermore, the expression profiles of Cry1 and pPer2 are synchronized but slightly staggered. This lag in time has previously been described in both the SCN and peripheral tissue [39], [51], supporting the concept of PER2 induced stabilization of CRY1 by prevention of ubiquitylation and proteosomal degradation [34], [52]. Together with the likewise adopted model of CRY1 induced stabilization of PER2 through similar mechanisms [52], [53], we propose a working model in which the hCRY1^AP^ protein enjoys increased stability leading to augmented pPER2 stability which downstream leads to increased stability of endogenous CRY1. Further supporting this view is the marked difference in expression amplitude between hCry1 and hCry1^AP^ transfected porcine fibroblasts.

Sleep-loss studies have identified physiological connection between the circadian clock and the immune system [54]. As a chronic state of metainflammation is known to be a substantial risk factor in metabolic disorders and cancer [55], [56], [57] and because the absence of CRY in knockout mice has been shown to entail increased expression levels of proinflammatory cytokines including TNF-α and IL-6 [58], we sought to determine if the antimorphic variant potentially could lead to a higher steady-state of inflammation in the transgenic animals. A considerable difference was apparent with average levels of IL-6, 4-fold (p = 0.047), and TNF-α, 5-fold (p = 0.0025), higher in fibroblasts from minipig #175-5^AP^ relative to the control. Notably, the oscillation of IL-6 in pig #175-5^AP^ displayed a periodicity of about 12 hours which is coherent with the expression profile of hCry1^AP^. However, IL-6 in the NT control followed the same pattern although pCry1 had a τ of about 24 hours.

CRY proteins have shown to bind adenylyl cyclase (ADCY) leading to a decrease in cyclic AMP production thereby preventing the activation of NF-κB and downstream expression of IL-6 - with the opposite effect taken place in the absence of CRY protein. Our data suggests that hCRY1^AP^ competes with endogenous CRY proteins for binding to ADCY but that it lacks repressor activity.

Body temperature (T_b_) control is a very important projection emanating from the SCN as it plays a role in the resetting of SCN itself but also as an auxiliary Zeitgeber of peripheral oscillators [51], [59]. Core T_b_ displays a clear circadian rhythm in mammals with fluctuations of 1–4°C [60]. We therefore wanted to assess whether the presence of hCRY1^AP^ protein would interfere with T_b_ control in the transgenic animals as previously observed in Cry1^−/−^/Cry2^−/−^ double knockout mice [61]. The average τ and amplitude of the T_b_ rhythm was unchanged (τ≈23 hours, p≤0.005) in the transgenic pigs compared to the controls under normal light-dark and feeding conditions. However, there were a few outliers and especially pig #208-4^AP^ presented an arrhythmic T_b_ oscillation, suggesting, that the central circadian clock, in at least this animal, is disturbed even under normal entrainment. It has previously been implied that CRY1 is dispensable for persistent SCN rhythmicity [37]. However, it is conceivable that the antimorphic hCRY1 mutant interferes with the circadian clock in a non-canonical fashion.

In summary we have generated 23 Bama-minipigs transgenic for a tissue unspecific antimorphic human Cry1. We have shown stable expression of the transgene in the skin of all cloned minipigs and demonstrated that this gives rise to altered circadian patterns of key peripheral oscillators as well as amplified induction of proinflammatory cytokines. Further analyses are currently undertaken and we anticipate that such porcine models will contribute to a better understanding of the link between the pathogenesis of circadian and metabolic disorders.

## Supporting Information

Figure S1 **Circadian rhythmicity in expression patterns of key circadian regulatory genes.** Fibroblasts expanded from skin-biopsies obtained from a non-transgenic minipig (#321-1) were serum-shocked after which total RNA was extracted every fourth hour through 52 hours. Quantitative RT-PCR was performed with exon-exon spanning primers targeting hCRY1, pPer2, pCry1, pClock, and pBmal1 normalized to endogenous ACTB. Expression is relative to the first measurement time point (ZT 0) and depicted as a function of time. The experiment is performed in triplicate and data are presented as means. Grey dashed lines indicate a complete 24 h oscillation (ZT 15 and 36 h, respectively).
(TIF)

Figure S2 **Comparison of acrophases of pCry1 and pPer2 expression in synchronized transgenic and non-transgenic epidermal fibroblasts.** The mRNA expression values of pPer2 and pCry1 depicted in figure 4 B–C were subdivided into windows of 12 or 24 hours. The acrophase was calculated using the free software program Acro (www.periodogram.org). The acrophase in ZT as well as the 95% confidence interval (CI) and a measure of goodness of fit is shown.
(TIF)

Table S1 Mean values and standard deviation (in brackets) of the relative mRNA expression levels shown in figure 4.
(TIF)

Table S2 One-way analysis of variance (ANOVA) comparing means of the relative mRNA expression level observed in the non-transgenic animal to the levels observed in the three transgenic animals as shown in figure 4. # Dunn's Multiple Comparisons Test (non-parametric analysis as SD's are not identical); ns not significant; * p<0.05; ** p<0.01; *** p<0.001.
(TIF)

Table S3 Chi-square periodogram data output (www.periodogram.org) using 51 body temperature entries per animal over a four day period.
(TIF)
