# Supplementary figures and images for: Development of Transgenic Minipigs with Expression of Antimorphic Human Cryptochrome 1
Source: PLoS One. 2013 Oct 16;8(10):e76098. doi: 10.1371/journal.pone.0076098 (PMC3797822; doi:10.1371/journal.pone.0076098)

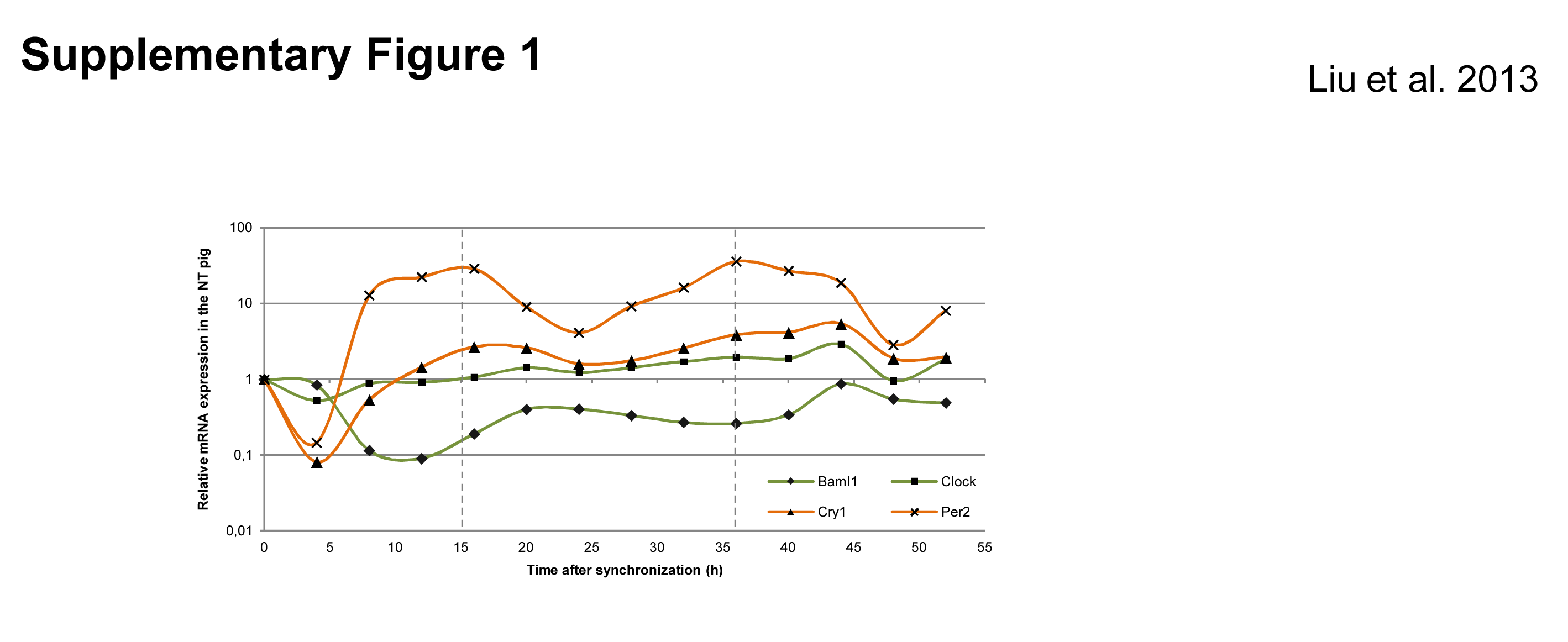

Supplement: Figure S1 — Circadian rhythmicity in expression patterns of key circadian regulatory genes. Fibroblasts expanded from skin-biopsies obtained from a non-transgenic minipig (#321-1) were serum-shocked after which total RNA was extracted every fourth hour through 52 hours. Quantitative RT-PCR was performed with exon-exon spanning primers targeting hCRY1, pPer2, pCry1, pClock, and pBmal1 normalized to endogenous ACTB. Expression is relative to the first measurement time point (ZT 0) and depicted as a function of time. The experiment is performed in triplicate and data are presented as means. Grey dashed lines indicate a complete 24 h oscillation (ZT 15 and 36 h, respectively). (TIF) [file pone.0076098.s001.tif]

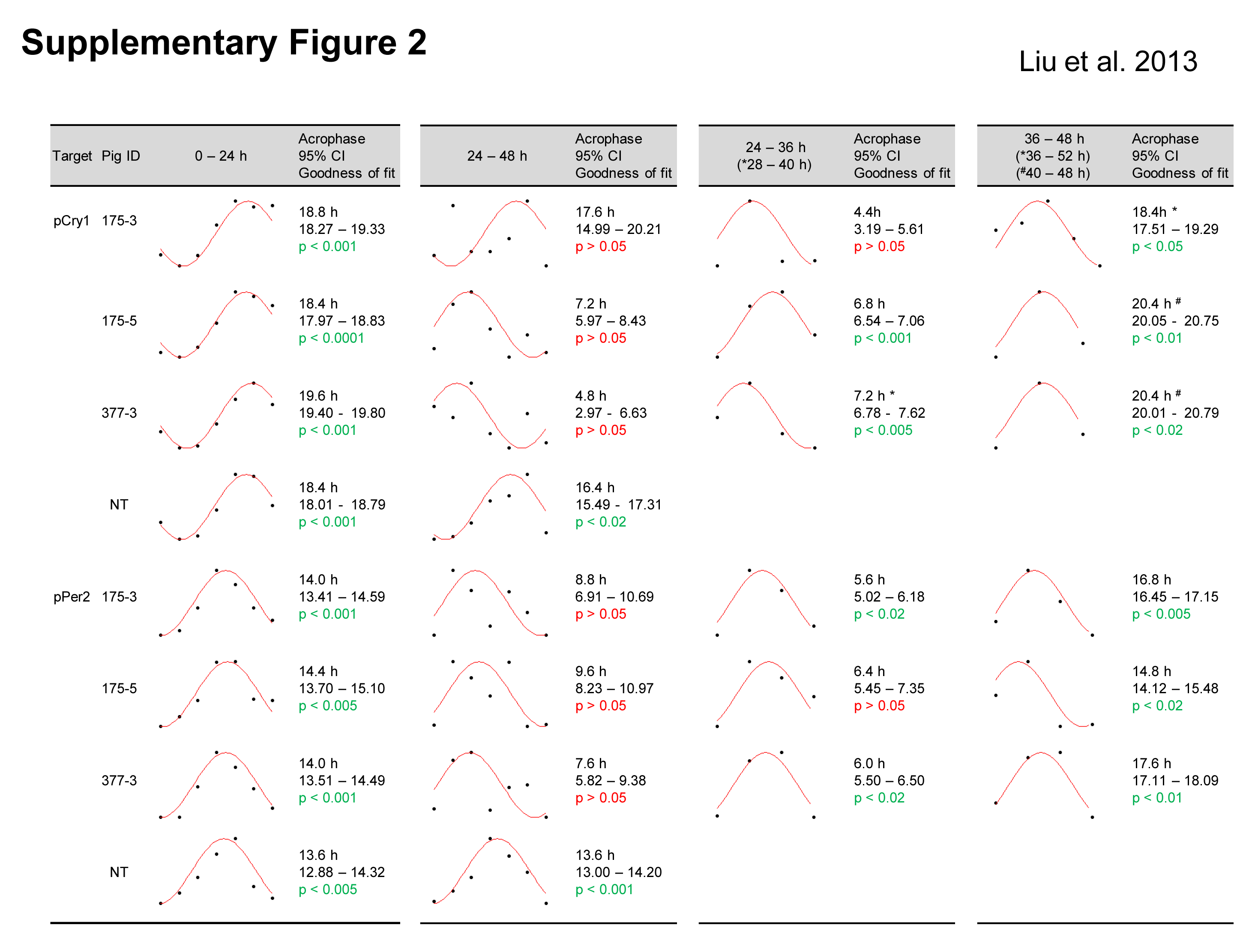

Supplement: Figure S2 — Comparison of acrophases of pCry1 and pPer2 expression in synchronized transgenic and non-transgenic epidermal fibroblasts. The mRNA expression values of pPer2 and pCry1 depicted in figure 4 B–C were subdivided into windows of 12 or 24 hours. The acrophase was calculated using the free software program Acro (www.periodogram.org). The acrophase in ZT as well as the 95% confidence interval (CI) and a measure of goodness of fit is shown. (TIF) [file pone.0076098.s002.tif]

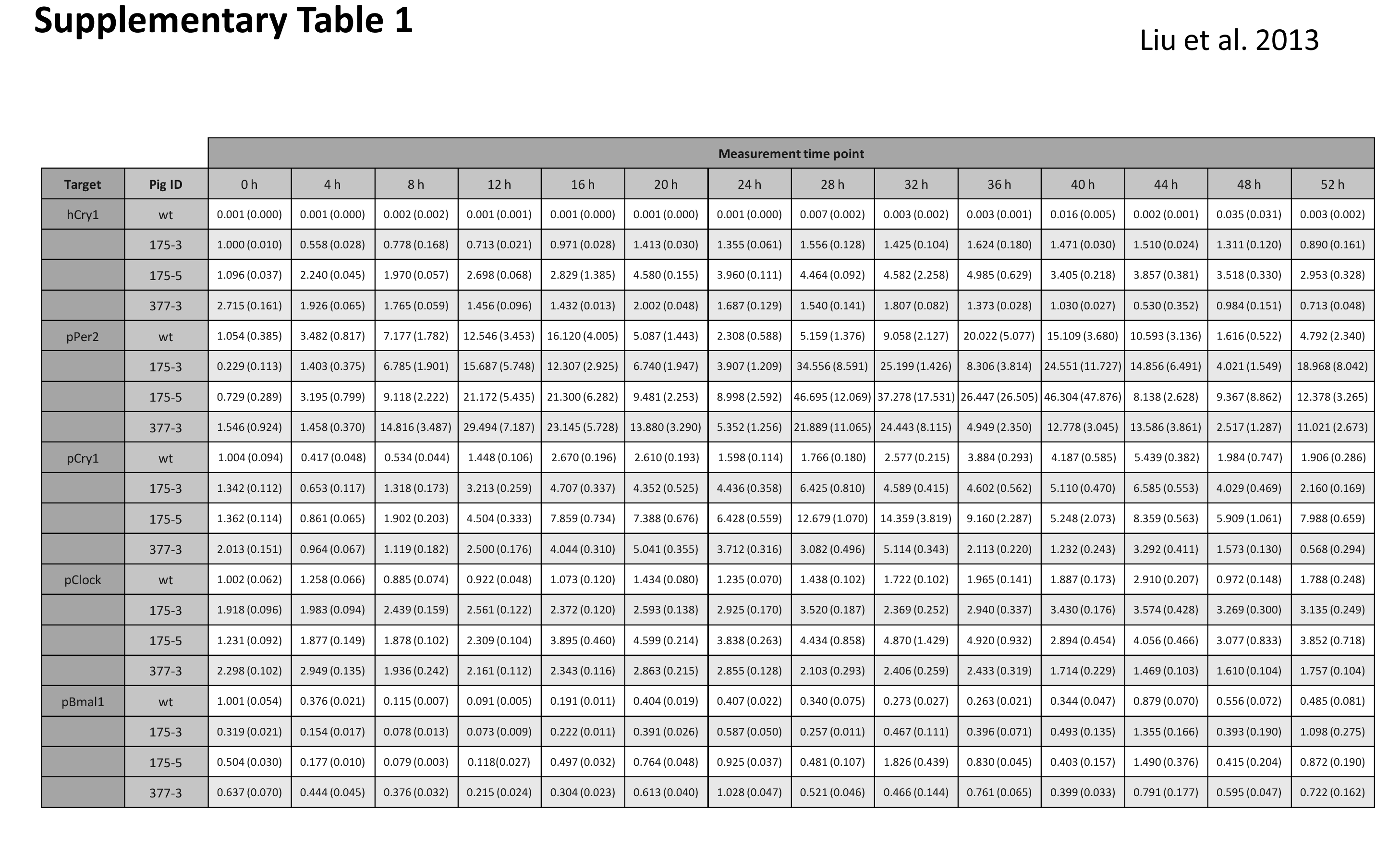

Supplement: Table S1 — Mean values and standard deviation (in brackets) of the relative mRNA expression levels shown in figure 4. (TIF) [file pone.0076098.s003.tif]

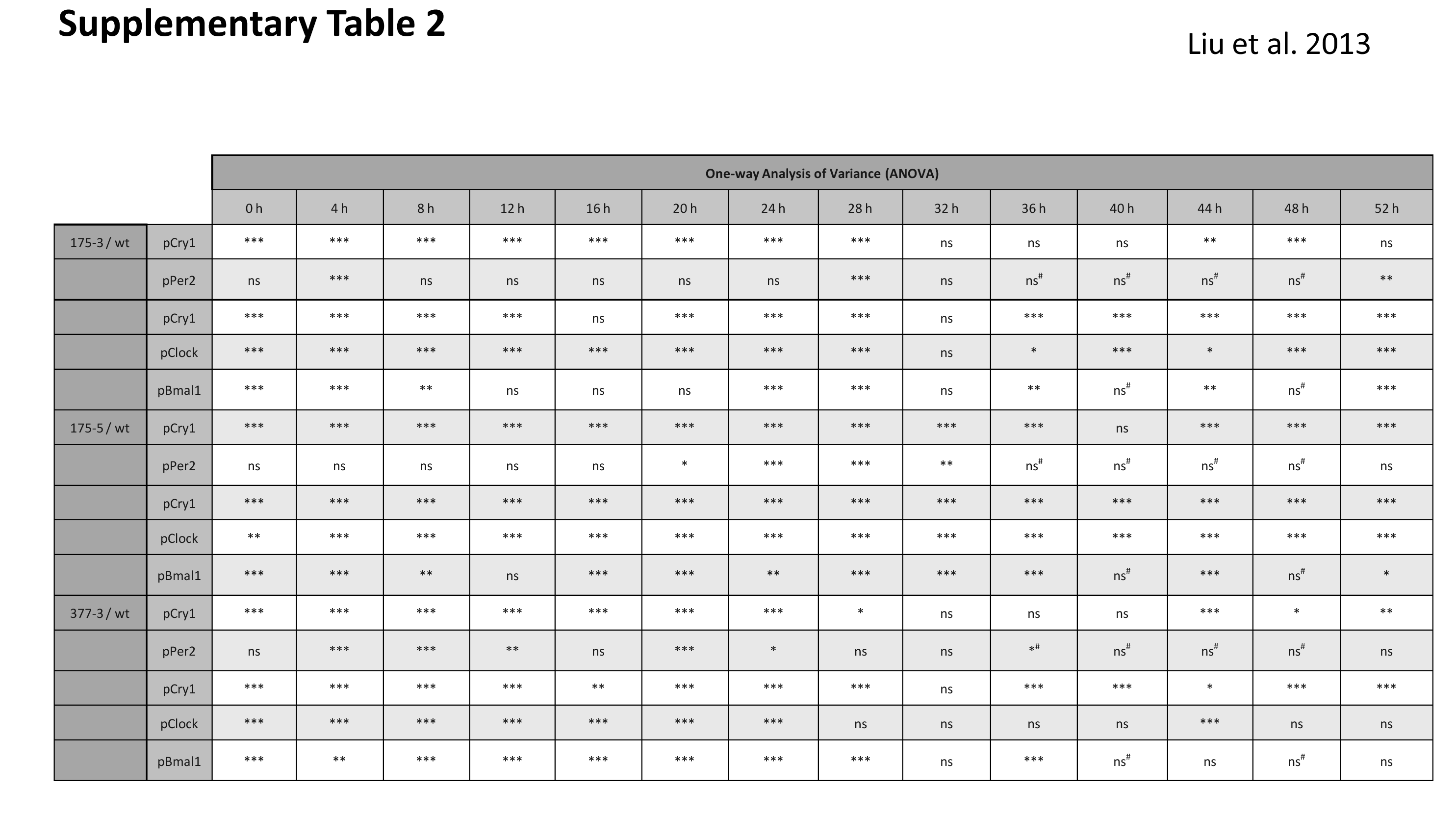

Supplement: Table S2 — One-way analysis of variance (ANOVA) comparing means of the relative mRNA expression level observed in the non-transgenic animal to the levels observed in the three transgenic animals as shown in figure 4. # Dunn's Multiple Comparisons Test (non-parametric analysis as SD's are not identical); ns not significant; * p<0.05; ** p<0.01; *** p<0.001. (TIF) [file pone.0076098.s004.tif]

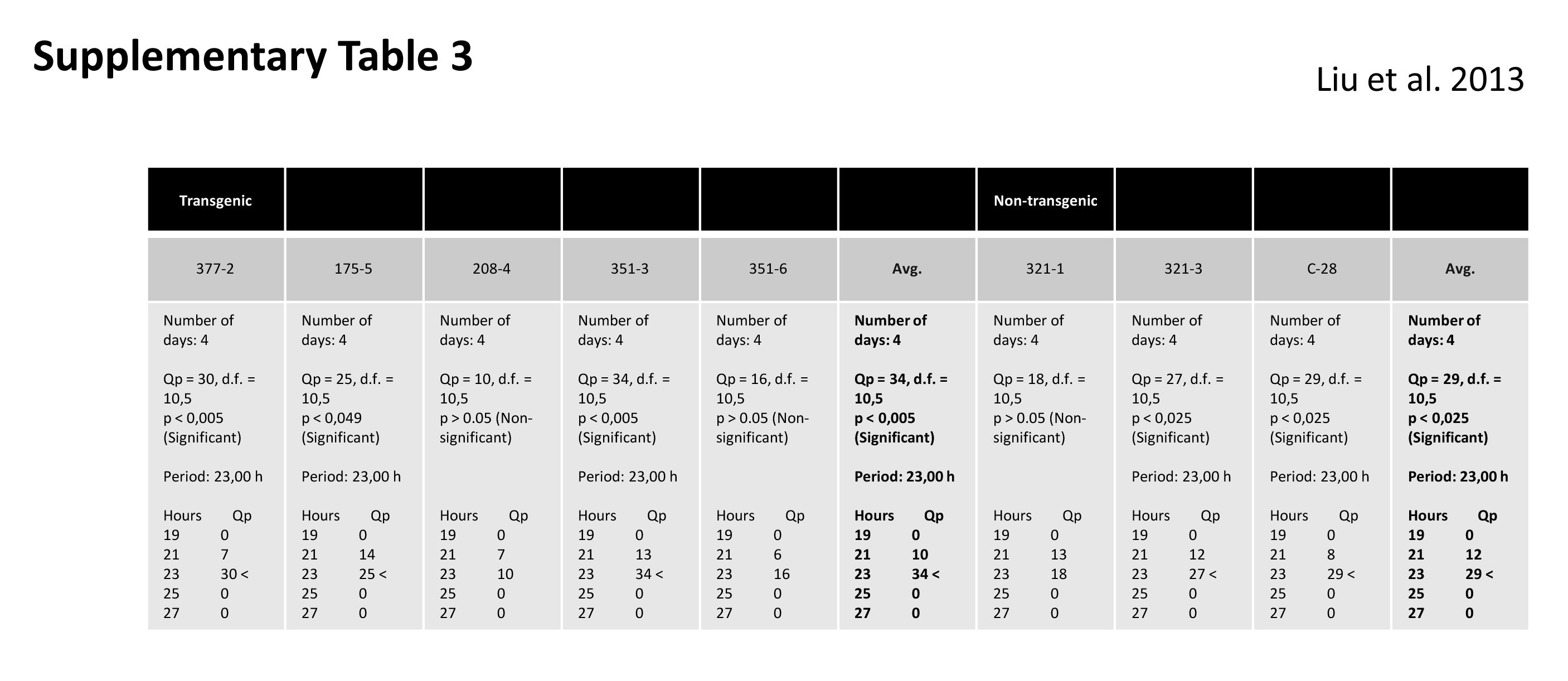

Supplement: Table S3 — Chi-square periodogram data output (www.periodogram.org) using 51 body temperature entries per animal over a four day period. (TIF) [file pone.0076098.s005.tif]
